# HIV disclosure status and factors among adult HIV positive patients in a secondary health facility in North-Eastern Nigeria, 2011

**DOI:** 10.11694/pamj.supp.2014.18.1.3551

**Published:** 2014-07-21

**Authors:** Raymond Salanga Dankoli, Alhaji A Aliyu, Peter Nsubuga, Patrick Nguku, Okechukwu P Ossai, Dahiru Tukur, Luka Ibrahim, James E. Madi, Mahmood Dalhat, Mohammed Abdullaziz

**Affiliations:** 1 Nigeria Field Epidemiology and Laboratory Training Programme (NFELTP), Abuja, Nigeria; 2 Department of Community Medicine, Ahmadu Bello University, Zaria, Nigeria; 3 Global Public Health Solutions, Decatur, 30033, GA, USA; 4 State Specialist Hospital, Gombe, Nigeria

**Keywords:** Disclosure Factors, HIV status, Gombe state, North-East, Nigeria

## Abstract

**Introduction:**

Disclosure of HIV status especially to sexual partners is an important prevention goal. This study was conducted to determine the prevalence of HIV status disclosure and the factors associated with disclosure by HIV positive patients attending the adult Anti-retroviral therapy (ART) clinic in State Specialist Hospital Gombe (SSHG) a secondary health facility in north-eastern Nigeria.

**Methods:**

We conducted a cross sectional study among adult HIV positive patients enrolled into the HIV/AIDS programme of SSHG. Study participant were sampled using a systematic random sampling. Interviewer administered questionnaire was used to collect data on socio-demographic characteristics, disclosure status and factors associated with disclosure. Data was analyzed using Epi-info software.

**Results:**

Of the 198 (99%) respondents, 159 (80.3%) were females. The mean age of respondents was 32.9years (SD ± 9.5). Sixty percent of the respondents were married. Most (97.5%) had disclosed their HIV status and majority (36.8%) disclosed to their spouses. Sixty four percent of the respondents had treatment supporter and spouses (42.9%) were their choice of a treatment supporter. Disclosure of HIV status was found to be associated with age < 40years Adjusted Odds Ratio (AOR) 38.16; 95% Confidence Interval (CI) 2.42-602.61. Gender, employment status, educational level, duration of infection and marital status were not found to be significantly associated with disclosure of HIV status.

**Conclusion:**

Disclosure of HIV status was high in the study population. Spouses were the most preferred choice of persons to disclose HIV status to, and the most adopted as treatment supporter. HIV status disclosure is encouraged after diagnosis because of its importance especially among couples.

## Introduction

HIV/AIDS represent the prototype of an emerging disease [1]. The Acquired Immune Deficiency Syndrome (AIDS) is caused by two lentiviruses; types 1 and 2 (HIV-1 and HIV-2) [2, 3]. AIDS was first recognized in the 1980’s and is presently the leading cause of death in developing countries, it is estimated that over 40million individuals have been infected with HIV of which about two-thirds live in sub-Saharan Africa [4].

The World Health Organization (WHO) and the United States Centers for Disease Control and Prevention (CDC) in their protocols for HIV testing and counseling emphasize the importance of disclosure of HIV status especially to sexual partners as an important HIV prevention goal [5, 6].

Disclosing HIV positivity status is a difficult and yet an important decision for all infected people [6, 7]. However, it has also been identified to play a significant role in how individuals cope with their disease [8, 9]. While disclosure can have positive effects on an individual’s health, such as increased social support and decreased stress, it also has the potential for negative effects which are dependent upon the recipient’s response. For the majority of individuals with HIV, disclosure is stressful, there is a fear of rejection, discrimination and abandonment, rather than the relief that is anticipated when one discloses to others [1, 10].

Sexual relationships within couples in sub-Saharan Africa is often associated with non-disclosure of HIV status to the individual partners, this can lead to sexual transmission of HIV within such stable relationships [11, 12]. Non-disclosure will discourage the adoption of safer sex practices among regular sex partners and their sexual relationships are rarely protected because they are perceived as risk free [11, 12].

HIV/AIDS treatment programmes now require that the HIV-infected person identifies a treatment supporter. Treatment supporters are individuals identified by each HIV positive patient to assist them comply with their clinic schedule, drug intake and other HIV related activities. These treatment supporters could be a spouse, a family member, a friend, a colleague or another person. A good and well informed treatment supporter encourages their client to adhere to the treatment protocol and follow up visits.

The HIV epidemic remains a public health concern and a threat to the socio-economic development of Gombe State in northeastern Nigeria, despite the persistent decline of adult prevalence. The dynamics, factors and drivers of the state’s epidemic are poorly understood because of dearth of strategic information [13].

Risky sex without disclosure of serostatus is not uncommon among people with HIV [14]. In the hospital where this study was conducted, doctors have received phone calls from husbands who are HIV positive and are receiving treatment pleading with them not to let their spouses know that they are HIV positive. This attitude demonstrates how individuals who are HIV positive can infect a series of unsuspecting partners. We therefore set out to determine the prevalence of HIV status disclosure and factors associated with disclosure of HIV status among HIV positive patients attending the adult anti-retroviral therapy (ART) clinic at the State Specialist Hospital Gombe (SSHG). The findings of this study will bridge the knowledge gap on issues of disclosure and non-disclosure of HIV status in Gombe state. Non-disclosure issues leading to loss to follow up will be identified and solution provided through client counseling. Through a feedback mechanism the HIV positive patients will be health educated on the need to adopt safer sex practices especially among couples when disclosure issues are properly addressed.

## Methods

We conducted a cross sectional survey of HIV positive patients who receive care and treatment at the SSHG between June 13 and July 17, 2011. SSHG is located in the capital city of Gombe state, northeastern Nigeria. It is the apex secondary health facility in the state and is a 300 bed capacity hospital and serves as a referral hospital to all state owned health facilities. In addition to other medical services, the hospital provides comprehensive HIV services which include voluntary counseling and testing (VCT), prevention of mother to child transmission (PMTCT), antiretroviral therapy (ART) for adult and children, support group services and treatment of opportunistic infections.

By 2011 there were more than 4,000 adult patients enrolled into HIV/AIDS care and treatment services of the hospital which started in 2006. Drugs and laboratory services were provided free to all HIV patients in the hospital.

We calculated the sample size for the study using the formula below:

n = z^2^xPx(1-P)/d^2^

Where n = sample size

Z = value of the standard distribution corresponding to a significant level of? (1.96 for a 2 sided test at 0.05 level type error level).

P = expected proportion in the population (Prevalence)

d = absolute precision which is significant at 0.05.

Since one of the factors associated with disclosure of HIV status is gender, we used the results from a previous study in Jos, Nigeria which showed a disclosure among women attending PMTCT clinic to be 89% [15].

Therefore n= 1.96^2^x0.89x0.11/0.05^2^ = 150.44 After addition of 10% non-response rate the total sample size used was approximated to 200.

A systematic random sampling method was used to select study participants. The adult ART clinic runs every Monday to Thursday with an average of 40 patients booked per clinic day. With a sample size of 200, the sampling interval of 5 was calculated by dividing 200 by 40.

The enrollment register of all the adult patients enrolled into the HIV programme of the hospital was used to select the participant using their unique ID number. The first participant was randomly selected from the booking register of each clinic day. Thereafter’> the sampling interval of 5 was calculated by dividing 200 by 40.

The enrollment register of all the adult patients enrolled into the HIV programme of the hospital was used to select the participant using their unique ID number. The first participant was randomly selected from the booking register of each clinic day. Thereafter, the next participant was selected after an interval of five numbers. After the selection process from the register was ‘completed’> the next participant was selected after an interval of five numbers. After the selection process from the register was ‘completed, the patients were identified based on their unique ID number at the clinic. The purpose of the study was explained to the identified participant and the study questionnaire was administered to those who consented.

Eligible participants were all individuals 16years and above who had been diagnosed as being HIV positive and had been enrolled into the hospital HIV/AIDS care programme and were attending the adult ART clinic.

Data were collected using a pre’> the patients were identified based on their unique ID number at the clinic. The purpose of the study was explained to the identified participant and the study questionnaire was administered to those who consented.

Eligible participants were all individuals 16years and above who had been diagnosed as being HIV positive and had been enrolled into the hospital HIV/AIDS care programme and were attending the adult ART clinic.

Data were collected using a pre-tested interviewer administered questionnaire. Five research assistants were recruited and trained on how to obtain informed consent from participants and how to improve response rate and also on how to effectively administer the questionnaire in order to obtain quality data. Data was edited, cleaned code and entered and analyzed using Epi-Info version 3.5.2 and Microsoft Excel. Descriptive statistics were calculated to determine rate of disclosure and other outcomes. Bivariate analyses were done to determine the presence of a statistically significant association between other independent variables and the HIV disclosure. Multiple logistic regression was conducted to control for confounding and to identify factors that were independently associated with disclosure.

All the dependent variables from the bivariate analyses were included in the logistic model. The logistic model coefficient were determined to be significant if their p value was ≤ 0.05 on the Wald Chi squared test.

Approval was received from the management of the SSHG to carry out the study. The purpose of the study was explained to each participant and a formal consent was signed by each participant before data were collected. Participation was voluntary and confidentiality was highly maintained.

## Results

Of the 200 respondents recruited for the study, 198 (99%) participated in the study. The majority (97.5%) of the participants had disclosed their HIV status. There were more female (80.3%) respondents than male. The mean age was 32.9 years (SD 9.5). The majority (76.8%) of the respondents were between 20’> 198 (99%) participated in the study. The majority (97.5%) of the participants had disclosed their HIV status. There were more female (80.3%) respondents than male. The mean age was 32.9 years (SD 9.5). The majority (76.8%) of the respondents were between 20-39 years old. Predominant tribes in the study were Fulani (24.7%), Tangale (21.7%) and Hausa (16.2%). Most of the respondents (55.7%) had below secondary education’> Tangale (21.7%) and Hausa (16.2%). Most of the respondents (55.7%) had below secondary education, while 28.3% of them were unemployed. The majority (60.1%) of the respondents were married (Table 1).

**Table 1:** Socio-demographic Profile of the HIV infected Study Participants, State Specialist Hospital, Gombe. 2011

Profile	Frequency(n = 198)	Percentage
**Age (years)**		
< 20	4	2.0
20-29	74	37.4
30-39	78	39.4
40-49	27	13.6
50-59	11	5.6
60 +	4	2.0
**Gender**		
Female	159	80.3
Male	39	19.7
**Educational Level**		
None	7	3.5
Arabic/Quaranic	72	36.4
Primary	31	15.7
Secondary	48	24.2
Tertiary	40	20.2
**Occupation**		
Student	4	2.0
Civil Servant	36	18.2
Farming	8	4.0
Business/Trading	45	22.7
Self employed	49	24.7
Unemployed	56	28.3
**Marital Status**		
Single	29	14.6
Married	119	60.1
Widowed	30	15.2
Separated	9	4.5
Divorced	11	5.6

In the bivariate analysis (Table 2) while 28.3% of them were unemployed. The majority (60.1%) of the respondents were married (Table 1)

**Table 2:** Association of HIV Disclosure factors and HIV Disclosure Status of Respondents at State Specialist Hospital, Gombe. 2011

Disclosure Factors		Disclosure Status		Total	Odds Ratios	95% Confidence Interval	P-value
		Yes (%)	No (%)				
**Marital Status**	Married	115 (96.6)	4 (3.4)	119	0.37	0.01-3.83	0.34
	Unmarried	78(98.7)	1(1.3)	79			
**Gender**	Female	156 (98.1)	3(1.9)	159	2.81	0.23-25.29	0.26
	Male	37 (94.9)	2 (5.1)	39			
**Educational Level**	<Secondary	36 (94.7)	2 (5.3)	38	0.64	0.07-7.94	0.47
	≥Secondary	85 (96.6)	3 (3.4)	88			
**Age**	<40years	155 (99.4)	1 (0.6)	156	16.31	1.53-808.43	0.008
	≥40years	38 (90.5)	4 (9.5)	42			
**Occupational Status**	Employed	127 (97.7)	3 (2.3)	130	1.28	0.11-11.47	0.56
	Unemployed	66 (97.1)	2 (2.9)	68	0.98	0.09-49.55	0.67
**Duration of Infection**	< 1 year	38 (97.4)	1 (2.6)	39			
	≥ 1 year	155 (97.5)	4 (2.5)	159			

In the bivariate analysis (Table 2), age > 40years old was significantly associated with disclosure of HIV status in the study population. Females OR 2.81; 95% Confidence Interval (CI) (0.23-25.29) those who were employed (OR 1.28; 95% CI 0.11-11.47) were more likely to disclose HIV status but this was not statistically significant. Gender, employment, duration of infection and marital status were not associated with HIV status disclosure.

We performed logistic regression to control for any confounding as shown in Table 3. The model included all factors that were analyzed at bivariate analysis. Age < 40years remained an independent factor in disclosure of HIV status both at bivariate and multivariate analysis, while female gender (AOR 1.53; 95% CI 0.14’> while female gender (AOR 1.53; 95% CI 0.14-17.02) and being employed (AOR 9.76; 95% CI 0.64-148.65) were not associated with HIV disclosure.

**Table 3:** Multivariate Analysis using Logistic Regression of Factors Associated with Disclosure of HIV Status among Study Participants State Specialist Hospital, Gombe 2011

Factor	Adjusted Odds Ratio	95% Confidence Interval	P-Value
Age group (<40years/≥40years)	38.2	2.4 - 602.6	0.01
Educational Status (Educated/Uneducated)	0.7	0.1 - 6.7	0.75
Gender (Female/Male)	1.5	0.1 - 17.0	0.73
Duration of infection time (<1year/≥1year)	0.2	0.0 - 3.2	0.24
Marital Status (Married/Unmarried)	0.3	0.0 - 4.2	0.38
Occupational Status (Employed/Unemployed)	9.8	0.6 - 148.7	0.10

**Figure 1:**
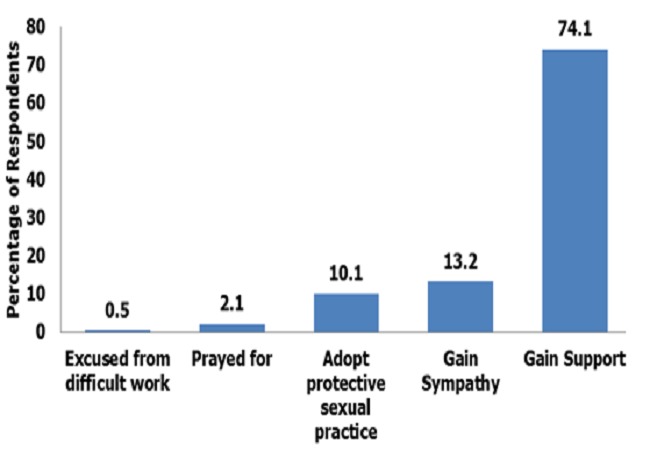
Reasons for HIV status disclosure among the respondents at State Specialist Hospital, Gombe, 2011

**Figure 2:**
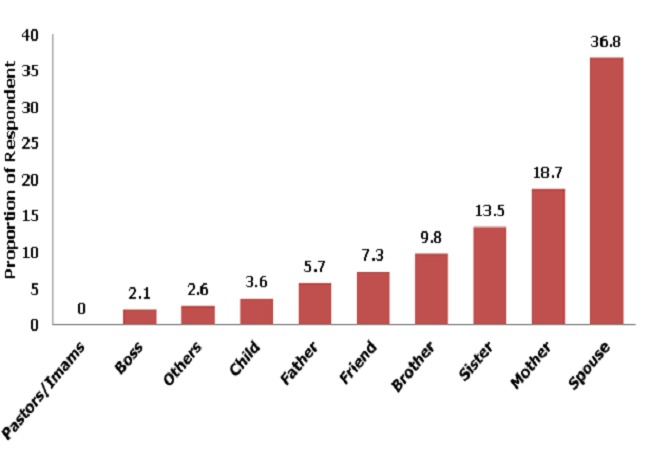
Preference of choice of first Person to disclose HIV status by the respondents at State Specialist Hospital, Gombe, 2011

Figure 1 shows reasons respondents said had led them to disclose their HIV status. These included to be excused from difficult work (0.5%),to be prayed for (2.1%)’> to be prayed for (2.1%), protective sexual practices (10.1%)’> to adopt protective sexual practices (10.1%), to gain sympathy (13.2%) and to gain support. (74.1%) which was the commonest reason cited. The most preferred choice of persons to disclose HIV status to by the respondents was their spouses (36.8%). Other female family relatives were next in the preferential choice’> to gain sympathy (13.2%) and to gain support. (74.1%) which was the commonest reason cited. The most preferred choice of persons to disclose HIV status to by the respondents was their spouses (36.8%). Other female family relatives were next in the preferential choice, mothers (18.7%)’> mothers (18.7%), and sisters (13.5%). Non family members were least preferred’> and sisters (13.5%). Non family members were least preferred-pastors/imams (0%), and boss (2.1%) (Figure 2).

**Figure 3:**
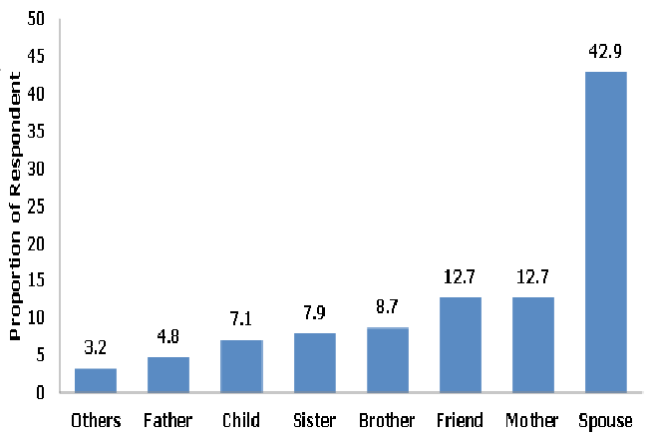
Choice of treatment supporter by the respondents at State Specialist Hospital, Gombe, 2011

The preference for spouses (42.9%) as treatment supporters was three times higher than preference for mothers (12.7%) and friends (12.7%) (Figure 3)

## Discussion

We found that the majority (97.5%) of respondents in our study of HIV-infected people in Gombe state had disclosed their HIV status. We also found out that the <40year old age group were more likely to have disclose their HIV status than the >40 years age group. In our study, participants preferred their spouses as the first person to disclose their status to and also the first choice for their HIV treatment supporter.

The high disclosure rate in our study is in agreement with the studies of Kim Bouillon et al [16] in the Caribbean region and that of Sagay AS, et al [15] in Northern Nigeria and Kebedeet al [17] in Southwest Ethiopia who reported HIV disclosure rates of 84.6%, 89% and 94.5% respectively. However, some studies reported lower disclosure rate, Issiakaeta [18] and Nebieet al [19] in Burkina Faso reported a disclosure rates of 31.8% and 17.6% respectively. The high disclosure rate in this study could be as a result of the ongoing adherence counseling sessions that encourage the HIV positive patients to disclose their status.

The study explored the influence of socio-demographic characteristics of the respondents on the disclosure attitude of HIV positive patients. There were more female (80.3%) than male (19.7%) among the study population. This is in consonant with the gender inequality prevalence of HIV in Gombe state where the prevalence amongst women was 4.8% and men was 2.2% [13]. Furthermore, according to the United Nation Human Development report on Nigeria, the burden of HIV infection in Nigeria is borne by young people with more females affected than males [20]. Gender was not a significant factor for HIV status disclosure in our study, however the study of Bouillon et al [15] at the Caribbean region shows male gender had lower disclosure rate. This is in contrast with a South Africa study that shows male were found to disclose their HIV status than female [21].

Age is an important factor in the spread of HIV/AIDS which mostly affect the productive age groups. The finding of this study supports the clustering of the respondent in the actively productive age group. About 75% of our respondents fell within the age bracket of 20-39years. Age was the only factor that was significantly associated with disclosure of HIV status in our study. Individuals with ages less 40years were more likely to disclose their HIV status than those who are 40years or more in the study population. Our findings are in consonant with the studies of both Farquhar et al [22] and Galliard et al [23] which showed that younger age group were more likely to disclose their HIV status than older age group. Age as a factor in HIV status disclosure is very important. That the older age group was less likely to disclosure their HIV status may be connected to the fact that they have been in a stable married relationship for a relatively long time, therefore, the knowledge of a partners HIV status may jeopardized the marriage relationship. Again, the younger age group may disclose their HIV status in order to assess anti-retroviral drugs early as this will help them to live longer.

Marital status was not significantly associated with HIV disclosure in our study however the married were less likely to disclose their HIV status compared to the unmarried. The implication of this is that, the HIV negative partners may be infected if disclosure is not done and unprotected sexual practice is adopted by couples.

In this study, majority of the respondent choose to disclose their HIV status because they wanted to gain the support of their confidant (Fig. 1) in managing their disease condition. This finding is similar to that of Kirshenbaum and Nevid,[24] Serovichet al[25] and Simoniet al[26] who found out that women who have lived longer with the disease are more likely to disclose their HIV status to obtain support surrounding their disease.

Regarding whom to disclose the HIV status to, most respondent prefer their spouses and this is in agreement with the study in University of Ilorin Teaching Hospital (UITH) Nigeria by Owolabiet al [27]. From our study and the UITH study there seems to be a paradigm shift from preferential disclosure to religious leaders as shown in the study by Daniel et al [28, 29]. in Sagamu Nigeria to disclosure to spouses and family members as found in our study (Fig. 2). In the same vein our study also showed that spouses were the most preferred choice as treatment supporters.

Generalizing the results of our study to other populations in Nigeria is limited by at least three issues. Firstly, data were self-reported and there was no mechanism in place to verify the information received from the respondents. Secondly, the study was hospital based and thus the results may not be directly applicable to the general population. Finally, the time for collection of data was only 6 weeks, which may limit generalization of our result since other patients could very well have different disclosure patterns although we have no reason to believe this is the case.

## Conclusion

Despite the common perception that people diagnosed with HIV often do not want to tell others about their status, majority of respondents in our study had disclosed their HIV status. Also, the preference of spouses as choice of whom to first disclose HIV status and use as treatment supporter is commendable. These findings will serve as a baseline data for further studies on related subject matter in the state.

Based on our study we recommend that the high disclosure rate should be maintained and improved upon. Relevant stakeholders should encourage furthering of education because the study showed that those with above secondary level of education are more likely to disclose their HIV status. Youth and women empowerment programmes of the Government will go a long way in making them socioeconomically stable. It is assumed that the more socioeconomically stable a person is, the more likely he/she will disclose his/her HIV status compared to the less socioeconomically stable. However, Farquhar et al found women of lower socioeconomic status had higher disclosure rate than women of higher socioeconomic status [22].

As the world is focusing on zero HIV new infection rate, HIV disclosure issues cannot be overemphasized. Nevertheless, HIV disclosure should be considered as a process rather than one time event. This study will form a base line data for further studies relating to HIV disclosure.
